# Book Review

**Published:** 1916-12

**Authors:** 


**Collosol Sulphur and Collosol Sulphur Cream.**—Messrs. Oppenheimer, Son & Company.—Colloidal preparations have come much into favour, and one of the latest developments of the Crookes laboratories in colloids is this sulphur colloid. It is especially recommended for rheumatic conditions, and where sulphur is considered of service is worthy of trial. The collosol sulphur liquid is a pleasant method of administering sulphur internally.

